# Sex-Dependent Prevalence of Sagittal Skeletal, Dental Malocclusions in Romanian Orthodontic Patients: An Observational Study

**DOI:** 10.3390/jcm15114011

**Published:** 2026-05-22

**Authors:** Bianca Maria Negruțiu, Bianca Ioana Todor, Cristina Paula Costea, Raluca Ortensia Cristina Iurcov, Ligia Luminița Vaida, Alexandra Ioana Lucan, Rebeca Lorena Gârboan, Claudia Judea Pusta, Marius Rus, Claudia Elena Staniș

**Affiliations:** 1 Department of Dental Medicine, Faculty of Medicine and Pharmacy, University of Oradea, 1st University Street, 410087 Oradea, Romania; 2 Biomedical Sciences Doctoral School, University of Oradea, 1st University Street, 410087 Oradea, Romania; 3 Târgu Mureș Emergency Clinical County Hospital, 50th Gheorghe Marinescu Street, 547530 Târgu Mureș, Romania; 4 Department of Morphological Disciplines, Faculty of Medicine and Pharmacy, University of Oradea, 1st University Street, 410087 Oradea, Romania; 5 Department of Medical Disciplines, Faculty of Medicine and Pharmacy, University of Oradea, 1st University Street, 410087 Oradea, Romania

**Keywords:** cephalometric analysis, sex, sagittal anomaly, orthodontics, digital orthodontics

## Abstract

**Objectives:** The present study aimed to evaluate the sexual dimorphism of skeletal and dental anomalies in Romanian orthodontic patients and to describe several important cephalometric measurements in patients with dental malocclusions. **Materials and Methods:** A total of 450 orthodontic records of patients older than 8 years were evaluated. On lateral cephalometric radiographs, the following cephalometric angles were digitally determined: SNA, SNB, ANB, FMA, IMPA, Max1-FH, SN-Go-Gn, N-A-Pog, Ar-Go-Me, and interincisal angle. The sagittal skeletal and dental malocclusions were diagnosed by two calibrated investigators. **Results:** The sample comprised 58% females, with a mean age of 20.07 (±8.63) years. The prevalence of dental malocclusions within the Romanian orthodontic sample taken into study was: 50.7% class I, 26.7% class II division 1, 13.3% class III, 4.7% class II, and class II division 2. The prevalence of skeletal anomalies within the Romanian orthodontic patient sample was: 43.3% class I, 28.7% class II due to retrognathic mandible, 17.3% class II due to prognathic maxilla, 8.7% class III due to prognathic mandible, and 2% class III due to retrognathic maxilla. Female patients presented more frequently with Class I or Class II division 2 malocclusion, whereas male patients more frequently exhibited Class III malocclusion. Female patients exhibited skeletal Class II more frequently due to retrognathic mandible, while skeletal Class III, due to prognathic mandible, was more common in male patients. Male patients were more frequently normodivergent, while female patients were more frequently hyperdivergent. Female patients exhibited retroclined upper incisors more frequently, whereas male patients exhibited proclined upper incisors more frequently. Most of the patients with class II division 1 malocclusion were females and exhibited the following cephalometric characteristics: a class II skeletal anomaly due to retrognathic mandible, normal SNA angle, decreased SNB angle, increased ANB angle, proclined upper incisors, proclined lower incisors, decreased interincisal angle, normal vertical growth pattern, closed mandibular angle, and convex facial profile. Most of the patients with class II division 2 malocclusion were females and exhibited the following cephalometric characteristics: a class II skeletal anomaly due to retrognathic mandible, normal SNA angle, decreased SNB angle, increased ANB angle, retroclined upper incisors, proclined lower incisors, increased interincisal angle, hypodivergent vertical growth pattern with a short face tendency, closed mandibular angle, and convex facial profile. Most of the patients with class III malocclusion were males and exhibited the following cephalometric characteristics: both class I and III skeletal anomaly due to prognathic mandible, normal SNA angle, increased SNB angle, decreased ANB angle, proclined upper incisors, normally inclined lower incisors, increased interincisal angle, hypodivergent, normal vertical growth pattern, and a short face tendency, normal mandibular angle, and balanced facial profile. **Conclusions:** The observed cephalometric differences between Class I, II and III malocclusions provide clinically relevant markers in vertical, sagittal, and dental dimensions that may provide descriptive reference data for similar orthodontic clinical samples.

## 1. Introduction

Malocclusion, the third-most prevalent oral health issue, is considered by some authors a complicated disorder determined by a combination of multiple factors such as genetics (excess or missing tooth, shape/size anomalies, jaw constriction, deep bite), congenital factors (dysostosis, cleft lip and/or palate), environmental factors (hormonal anomalies, avitaminosis, TMJ disorders, sucking habits, impaired nasal breathing), ethnic factors, functional atrophy of the maxilla [1,2,3,4]. Others consider that malocclusion is not actually a disease, but a set of various levels of deviations from optimal occlusion [5].

Effective prevention, accurate diagnosis, early intervention and control of the malocclusion can be achieved only by a thorough comprehension of how the disease occurs, its distribution and the factors that influence its development within a certain population [6,7,8]. Thus, cephalometry is an essential diagnostic tool when assessing patients’ face, dental and skeletal morphological features [9]. Patients’ skeletal class can be determined by several methods, ranging from traditional approaches such as the ANB angle described by Riedel [10] to individualized techniques including Fishman’s graphical method [11], the harmony box of Segner and Hasund [12], and the individualized ANB proposed by Panagiotidis and Witt [13].

Skeletal Class II pattern is typically described as a discrepancy in the anteroposterior relationship between the maxilla and the mandible (ANB greater than 4°), characterized by a relatively protruded maxilla (maxillary protrusion or prognathism), a retruded mandible (mandibular retrognathism), or a combination of both conditions [14,15,16].

Skeletal Class III pattern is often easily recognized clinically due to the mandibular protrusion associated with an anterior crossbite. Dental Class III malocclusion is not characterized by skeletal discrepancy. In contrast, skeletal Class III involves a range of skeletal and dental patterns (ANB less than 0°): protrusion of the mandible (mandibular prognathism), retrusion of the maxilla (maxillary retrognathism) or a combination of both conditions [17,18,19].

Considering Angle’s classification system based on the sagittal first molars relationship, patients’ dental malocclusions can be categorized as Class I, Class II, and Class III. Class I, often defined as normal occlusion, is characterized by the placement of the mesiobuccal cusp of the maxillary first molar in the buccal groove of the mandibular first molar and, according to Andrews, by a proper marginal ridge relationship between the distal marginal of the maxillary first molar and the mesial marginal ridge of the lower second molar. Class II, characterized by the placement of the mesiobuccal cusp tip of the upper first molar in contact with the embrasure space between the lower first molar and second bicuspid (distal), is further divided into Class II Division 1, Class II Division 2 and Class II. Class III is defined by a contact between the mesiobuccal cusp tip of the upper first molar and the embrasure space between the lower first and second molars (mesial) [15,20].

Along with maxillary and mandibular morphology and position, both upper and lower incisors’ position and inclination play an important role in achieving a harmonious and balanced facial profile. Therefore, determining the position of the incisors should also be evaluated when considering the camouflage of underlying skeletal anomalies [21,22].

These dental and skeletal anomalies, along with enamel defects like molar-incisor hypomineralization, have a considerable impact on both health and quality of life [23,24]. It is proven that individuals with Class II and III malocclusion tend to experience poorer psychological, social and physical quality of life compared to those with Class I malocclusion [25].

Moreover, an accurate prevalence of dental and skeletal malocclusions is essential for estimating the magnitude of health problems [8]. Malocclusion in children and adolescents is highly prevalent worldwide, affecting approximately one in two individuals or more [26]. Class II malocclusion is more prevalent than Class III malocclusion, with reported prevalence ranging from 5% to 29% [27]. Additionally, nearly 66% of Class II division 1 patients present a significant skeletal discrepancy [28]. However, most available studies are based on samples of patients attending orthodontic clinics, which may lead to an overestimation of malocclusion prevalence compared with the general population [29].

Several authors recommend considering ethnic differences in orthodontic practice and suggest using population individualized standards for accurate diagnosis [26,30]. Skeletal class II anomalies represent more than one-third of all malocclusions globally and are more commonly observed in Caucasian populations compared with other ethnic groups [15]. In contrast, the frequency of Class III malocclusion varies in the general population from 4% among Caucasians to 14% among Asians [31]. Class III malocclusion is reported to be more prevalent in Middle Eastern populations than in Caucasians, but less common than in East Asian populations [32].

Therefore, an accurate diagnosis of skeletal Class II patients, in comparison with an ideal skeletal Class I relationship, is of key importance for orthodontic practitioners [19].

Considering the multifactorial etiology of dental malocclusion, the distinct morphological features characterizing each type of skeletal anomaly, and the limited body of literature addressing these aspects, the present study aimed to determine the sexual dimorphism of the skeletal and dental anomalies in Romanian orthodontic patients and to describe several important cephalometric measurements in patients with dental malocclusions. Hence, this study seeks to contribute to the existing body of literature by offering an evaluation of cephalometric measurements in Romanian orthodontic patients. Furthermore, it may hold clinical relevance by serving as a practical reference for orthodontists in the selection of the most appropriate treatment strategies for their patients.

To our knowledge, no study has systematically compared cephalometric parameters across different subgroups of Romanian orthodontic patients exhibiting skeletal Class II or Class III malocclusions. In addition, variations related to sex have not been comprehensively investigated in relation to skeletal anomalies within a Romanian orthodontic sample.

The following null hypotheses were tested in this study:

- There is no significant association between sex and the type of dental/skeletal malocclusion.
- There is no significant association between the type of dental/skeletal malocclusion and various cephalometric measurements such as FMA, SNA, SNB, ANB angles, etc.

## 2. Materials and Methods

### 2.1. Ethical Considerations

This study was conducted in accordance with the ethical principles outlined in the 1964 Declaration of Helsinki and its subsequent amendments. Ethical approval was granted by the Research Ethics Committee of the University of Oradea (Approval no. 323/3 November 2025). Informed consent to use their medical records for research purposes was obtained from all participants or their legal guardians prior to enrolment and treatment. Before agreeing to be included in this study, participants were informed that their involvement was entirely voluntary and anonymous, and that no financial or other incentives were offered.

### 2.2. Sample Selection

This investigation was designed as a retrospective observational study and is reported in accordance with the STROBE (Strengthening the Reporting of Observational Studies in Epidemiology) guidelines for cross-sectional studies. This study was conducted between November 2025 and February 2026 in two orthodontic settings located in Oradea, Romania: The University Dental Clinic affiliated with the Faculty of Medicine and Pharmacy at the University of Oradea and a private orthodontic practice collaborating with the university. Available for this study was a total of 523 records of orthodontic patients who were patients in the two settings during the last 5 years. The same research team supervised data collections across both sites to ensure methodological consistency.

### 2.3. Inclusion and Exclusion Criteria

Patients 8 years of age and older, whose dental records included initial orthopantomography, lateral cephalometric radiographs of good quality, intraoral and extraoral photos, and study casts, were taken into the study. The maximum age limit of the sample was 48 years, and all of the orthodontic files had a complete dental history of the patient.

Inclusion criteria comprised patients with various types of dental and skeletal anomalies who were willing to undergo orthodontic treatment. Eligible participants were non-syndromic, without craniofacial deformities, cleft lip and/or palate, or a history of prior orthodontic treatment. So, exclusion criteria comprised patients who presented a history of trauma, cleft lip and/or palate, syndromes, endocrine imbalances and/or metabolic disorders, and prior orthodontic treatment.

**Figure 1:**
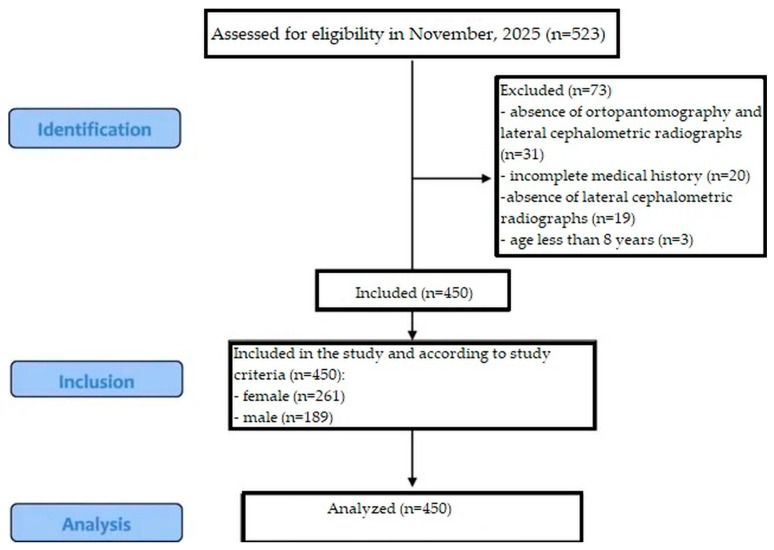
Strobe participant flow diagram.

After the inclusion and exclusion criteria were applied, the final sample consisted of 450 patient records. From the total of 523, 73 were excluded because of the absence of orthopantomography and lateral cephalometric radiographs (42.5%), incomplete medical history (28.4%), absence of lateral cephalometric radiographs (25%) and age less than 8 years (4.1%) (Figure 1).

### 2.4. Characterization of Growth Pattern and Skeletal Classification

All lateral radiographs were taken before the orthodontic treatment in the natural head position with posterior teeth in maximum intercuspation.

To characterize growth patterns, several cephalometric parameters were initially digitally measured using the OnyxCeph^3^™ v. 3.2 program (Image Instruments GmbH, Chemnitz, Germany) by the same investigator (Table 1).

### 2.5. Diagnosis of Dental Malocclusions

Using the initial panoramic and lateral radiographs, photos and study casts, five dental malocclusions were defined:

- Class I;
- Class II;
- Class II division 1;
- Class II division 2;
- Class III.

Class I malocclusion is defined by the occlusion of the mesiobuccal cusp of the maxillary first permanent molar with the mesiobuccal groove of the mandibular first permanent molar. Class II malocclusion is characterized by a mesial positional relationship of the mesiobuccal cusp of the maxillary first molar relative to the mesiobuccal groove of the mandibular first molar. It is further divided into Class II Division 1 (skeletal class II anomaly combined with proclined upper incisors), Class II Division 2 (skeletal class II anomaly combined with retroclined upper incisors) and Class II (skeletal class II anomaly combined with normally inclined upper incisors). In contrast, Class III malocclusion is defined by a distal positional relationship of the mesiobuccal cusp of the maxillary first permanent molar relative to the mesiobuccal groove of the mandibular first permanent molar.

Information about age and sex was also collected from the dental records. All clinical data was collected by a single calibrated investigator.

### 2.6. Reliability

For each patient, the following standardized workflow was implemented:

- Initial collection of clinical data, including age and sex, from the dental records;
- Each examiner received an individual folder for each patient included in the study. The folder contained the patient’s age and sex, the initial panoramic radiograph, intraoral and extraoral photographic records, and the initial lateral cephalometric X-ray. In addition, the corresponding study cast was provided in physical format for direct evaluation.
- Assessment of the initial panoramic radiographs and photographic records, as presented in the present document;
- Evaluation of the study casts, which were physically examined by the investigators;
- Cephalometric analysis of the lateral radiographs using three analytical methods (Riedel, Roth–Jarabak, and Tweed), performed by the first examiner;
- Recording of the obtained measurements and observations in a dedicated Excel database by the first examiner;
- Independent cephalometric analysis of the lateral radiographs using the same three analytical methods (Riedel, Roth–Jarabak, and Tweed), performed by the second examiner;
- Recording of the obtained measurements and observations in a dedicated Excel database by the second examiner;
- Reassessment of the cephalometric radiographs after a 25-day interval by the first examiner using the same three analytical methods, with data entered into a separate Excel database in order to minimize measurement bias and examiner influence;
- Reassessment of the cephalometric radiographs after a 25-day interval by the second examiner using the same three analytical methods, with data recorded in a separate Excel database to minimize potential examiner-related bias;
- Consolidation and centralization of all collected data into a single Excel database;
- Statistical analysis of the compiled dataset.

For the diagnosis of dental anomalies and values of the SNA, SNB, ANB, SN-GoGn, FMA, IMPA, Max1-FH, Ar-Go-Me, interincisal, N-A-Pog angles, reliability was assessed by a gold standard evaluator, a specialist in orthodontics with more than 10 years of experience. The gold standard evaluator performed the diagnosis of 450 individuals using the panoramic radiographs, study casts (to assess the dental anomalies) and the lateral cephalometric radiographs (to assess the angles), with the aid of a negatoscope and the OnyxCeph^3^™ v. 3.2 program (Image Instruments GmbH, Chemnitz, Germany). Subsequently, another evaluator performed the same diagnosis under the same conditions to compare the results. In an interval of 25 days, a repetition of these same steps was performed by the investigators to obtain interexaminer reliability statistics. Intra-rater reliability was measured for rater 1 and rater 2 between the two measurements, while inter-rater reliability was measured between raters for the first measurement. For nominal variables (dental malocclusion and skeletal anomalies), reliability was measured using Cohen’s kappa. For cephalometric angles, intraclass correlation coefficients (ICC (3,1)) with a two-way mixed design and absolute agreement were calculated with 95% confidence intervals.

### 2.7. Statistical Analysis

Data analysis was conducted using IBM SPSS Statistics v25 (IBM Corp., Armonk, NY, USA) and results were created in Microsoft Office Excel and Word 2024 (Microsoft Corp., Redmond, WA, USA). Quantitative variables were expressed as means with standard deviations or as medians with interquartile ranges, while qualitative variables were reported as frequencies and percentages. Differences between groups were tested using Fisher’s Exact Test. Z-tests with Bonferroni correction were used to further detail the results obtained in the contingency tables. The threshold considered for the significance level for all tests was considered to be α = 0.05.

## 3. Results

Based on the inclusion and exclusion criteria, the final study sample consisted of 450 orthodontic patients. Regarding age distribution, 30 patients (6.7%) were aged 8–12 years, 228 patients (50.7%) were aged 13–18 years, and 192 patients (42.7%) were older than 18 years. In terms of sex, the majority were female (n = 261, 58%), while male patients accounted for 42% (n = 189) (Table 2).

The most frequent dental and skeletal malocclusion was Class I (50.7%, 43.3%). Most patients showed a normal SNA angle (n = 180, 40%), while 117 patients (26%) showed a decreased SNA angle and 153 patients (34%) showed an increased SNA angle. Most patients exhibited a decreased SNB angle (n = 186, 41.3%), while 32.7% exhibited a normal SNB angle (n = 147 patients) and 26% (n = 117) exhibited an increased SNB angle. The majority of patients showed an increased ANB angle (52%, n = 234), while 35.3% (n = 159) showed a normal ANB angle and 12.7% (n = 57) showed a decreased ANB angle.

Most of the patients exhibited proclined upper (n = 222, 49.3%) and lower incisors (n = 189, 42%). Regarding the vertical growth pattern of the orthodontic patients, the number of hypodivergent patients (n = 186, 41.3%) was almost equal to the number of normodivergent patients (n = 189, 42%). Moreover, the number of patients with a closed mandibular angle (n = 201, 44.7%) was almost equal to the number of patients with a normal Ar-Go-Me angle (n = 216, 48%).

Table 3 shows the distribution of the patients according to dental malocclusion and age. Differences between groups were significant (***p* < 0.001**). Z-tests with Bonferroni correction show that patients exhibiting class I malocclusion were more frequently aged 13–18 years or ≥18 years rather than 8–12 years (56.6%/50% vs. 10%), patients with class II/2 malocclusion were more frequently aged 13–18 years rather than ≥18 years (9.2% vs. 0%) and patients with class III malocclusion were more frequently aged 8–12 years rather than 13–18 years or ≥18 years (60% vs. 5.3%/15.6%).

Table 4 summarizes the distribution of patients by sex and several cephalometric parameters. Dental malocclusions, skeletal anomalies and FMA angle were significantly different according to sex (***p* < 0.001**). Post hoc Z-tests with Bonferroni correction showed that:

- Female patients presented more frequently with Class I (55.2% vs. 44.4%) or Class II division 2 malocclusion (6.9% vs. 1.6%), whereas male patients exhibited Class III malocclusion more frequently (23.8% vs. 5.7%).
- Female patients exhibited skeletal Class II more frequently due to retrognathic mandible (36.8% vs. 17.5%), while skeletal Class III due to prognathic mandible was more common in male patients (14.3% vs. 4.6%).
- Male patients were more frequently normodivergent (47.6% vs. 37.9%), while female patients were more frequently hyperdivergent (23% vs. 7.9%).
- The inclination of the maxillary incisors (Max1-FH angle) was significantly different according to sex (***p* = 0.003**). Post hoc Z-tests with Bonferroni correction showed that female patients exhibited retroclined upper incisors more frequently compared to males (19.5% vs. 9.5%), whereas male patients exhibited proclined upper incisors more frequently (57.1% vs. 43.7%).

Table 5 shows that dental malocclusion distribution by age is not significantly different (*p* = 0.457), while according to gender remains significant (*p* < 0.001). Z-tests with Bonferroni correction show that patients with class I malocclusion were significantly more frequently females (55.2% vs. 44.4%), while patients exhibiting class III malocclusion were significantly more frequently males (23.8% vs. 5.7%).

Table 6 shows that while adjusting for age (which had no significant influence), gender remains a significant predictor for dental malocclusion. This supports the idea that female patients show higher odds than male patients to exhibit a class I dental malocclusion by 5.319 times (95% C.I. = 2.777–10.101) (*p* < 0.001).

Table 7 describes the comparison of cephalometric measurements according to dental malocclusion. Results show the following (considering also Z-tests with Bonferroni correction and post hoc Dunn-Bonferroni tests):

- Age was significantly different across groups (*p* < 0.001): patients aged 8–12 exhibited Class III malocclusion more frequently (30% vs. 7.5%/0%), patients aged 13–18 showed more frequently Class II/2 malocclusion (100% vs. 47.5%/20%) and patients over 18 years old exhibited more frequently Class II/1 or Class III malocclusion (45%/50% vs. 0%);
- Gender was significantly different across groups (*p* < 0.001): female patients showed more frequent class II/1 or class II/2 malocclusion (57.5%/85.7% vs. 25%), while male patients showed more frequent class III malocclusion (75% vs. 42.5%/14.3%);
- Skeletal anomalies were significantly different across groups (*p* < 0.001): patients with Class I skeletal anomaly showed more frequently Class II/1 or class III dental malocclusion (25%/45% vs. 0%), patients with Class II skeletal anomaly due to prognathic maxilla showed more frequently class II/1 or class II/2 dental malocclusion (25%/28.6% vs. 0%), patients with class II skeletal anomaly due to retrognathic mandible showed more frequently class II/1 or class II/2 dental malocclusion (50%/71.4% vs. 0%), patients with class III skeletal anomaly due to retrognathic maxilla showed more frequently class III dental malocclusion (10% vs. 0%/0%) and patients with classs III skeletal anomaly due to prognathic mandible showed more frequently class III dental malocclusion (45% vs. 0%/0%);
- SNA was significantly different across groups (*p* = 0.033): patients with class II/2 malocclusion had higher SNA values than patients exhibiting class II/1 malocclusion (*p* = 0.029);
- ANB was significantly different across groups (*p* < 0.001): patients with class III malocclusion had lower ANB values than patients exhibiting class II/1 (*p* < 0.001) or class II/2 malocclusion (*p* < 0.001);
- SNB was significantly different across groups (*p* < 0.001): patients with class III malocclusion had higher SNB values than patients exhibiting class II/1 (*p* < 0.001) or class II/2 malocclusion (*p* < 0.001);
- S-N/Go-Gn was significantly different across groups (*p* = 0.001): patients with class II/2 had lower S-N/Go-Gn values than patients exhibiting class II/1 malocclusion (*p* = 0.001);
- N-A-Pog was significantly different across groups (*p* < 0.001): patients with class III malocclusion had lower N-A-Pog values than patients exhibiting class II/1 (*p* < 0.001) or class II/2 malocclusion (*p* < 0.001);
- Interincisal angle was significantly different across groups (*p* < 0.001): patients with class II/2 malocclusion had higher interincisal angle values than patients exhibiting class II/1 (*p* < 0.001) or class III malocclusion (*p* = 0.002). Also, patients with class III malocclusion had higher interincisal angle values than patients exhibiting class II/1 malocclusion (*p* < 0.001);
- Max1-FH was significantly different across groups (*p* < 0.001): patients with class II/2 malocclusion had lower Max1-FH values than patients exhibiting class II/1 (*p* < 0.001) or class III malocclusion (*p* < 0.001);
- Ar-Go-Me was significantly different across groups (*p* < 0.001): patients with class II/2 malocclusion had lower Ar-Go-Me values than patients exhibiting class II/1 (*p* < 0.001) or class III malocclusion (*p* < 0.001). Also, patients with class III malocclusion had higher Ar-Go-Me values than patients exhibiting class II/1 malocclusion (*p* < 0.001);
- FMA was significantly different across groups (*p* = 0.002): patients with class II/2 malocclusion had lower FMA values than patients with class II/1 (*p* = 0.001) or class III malocclusion (*p* = 0.027);
- IMPA was significantly different across groups (*p* < 0.001): patients with class III malocclusion had lower IMPA values than patients exhibiting class II/1 (*p* < 0.001) or class II/2 malocclusion (*p* < 0.001).

Overall, patients with class III malocclusion showed lower ANB, N-A-Pog, IMPA and higher SNB than class II/1 or class II/2 groups. Patients with class II/2 malocclusion exhibited higher SNA, interincisive angle, lower S-N/Go-Gn, Max1-FH, Ar-Go-Me and FMA than patients with class II/1 malocclusion.

## 4. Discussion

According to the World Health Organization (1987), malocclusion is defined as an abnormality that may result in disfigurement or functional impairment and which requires treatment when it constitutes an obstacle to an individual’s physical or mental well-being, significantly affecting self-esteem and social acceptance [33,34].

This study aimed to investigate differences in several important cephalometric variables with respect to sex, dental, and skeletal anomalies using multiple comparison tests, and to further explore the correlations among various cephalometric parameters within a specific Romanian orthodontic sample. Individualized cephalometric methods provide descriptive reference data for similar orthodontic samples. However, interpretation of the results should account for possible variations related to age, sex, and population-specific characteristics [35].

The study group shows some individualized characteristics. With respect to age distribution, the majority of patients in the study group were aged 13–18 years. During adolescence, individuals become increasingly aware of their physical irregularities and, consequently, tend to place greater emphasis on personal care and appearance, as previously reported in the literature [36].

Considering sex distribution, the majority of patients in the study group were females. This finding may be explained by the greater tendency of females, compared to males, to seek orthodontic treatment in order to improve facial aesthetics and overall appearance, as previously reported in the literature [37].

Based on our results, Romanian orthodontic patients can be characterized by the following cephalometric and dental features: a predominance of Class I dental occlusion, followed by Class II division 1 and Class III malocclusions; a predominance of Class I skeletal pattern, followed by Class II due to retrognathic mandible and Class II due to prognathic maxilla; a predominantly normal SNA angle, closely followed by increased values; a decreased SNB angle, followed by normal values; an increased ANB angle, followed by a normal values; proclined upper and lower incisors; a hypodivergent growth pattern nearly equal in frequency to normodivergent pattern; and a predominantly closed mandibular angle, with a frequency comparable to that of normal mandibular angle values.

Skeletal Class II malocclusion comprises more than one-third of malocclusion cases worldwide and is observed more commonly among Caucasian populations [15]. Concerning the predominance of Class II division 1 malocclusion, our findings are consistent with those reported by De Ridder et al. (2022), but are in contrast to the results of Atasever et al. (2025), who identified a higher prevalence of Class II division 2 compared with Class II division 1 malocclusion. This discrepancy may be attributed to the increased prevalence of overbite and deep bite observed in the population examined by Atasever et al. (2025) [38,39]. Similar to studies conducted in other populations, including Iranian, Dutch, Nigerian, Libyan, and Egyptian cohorts, the Turkish population, similar to the Romanian population, has been reported to exhibit a predominance of Class I malocclusion, followed by Class II and Class III malocclusions [40,41,42,43,44].

Concerning the prevalence of Class III dental and skeletal malocclusion, the present study identified rates of 10.7% for skeletal Class III and 13.3% for dental Class III within the Romanian orthodontic sample taken into study. In the Chinese and Saudi Arabian populations, Class I malocclusion demonstrated the highest estimated frequency, followed by Class III malocclusion, while Class II malocclusion showed a markedly lower frequency [45,46]. The higher occurrence of Class III malocclusion reported in these populations may be explained by variations in craniofacial structure across ethnic groups, underlying genetic factors, and environmental influences, in addition to differences in study design, including age range and sample size [47]. However, several previous studies have reported a considerably lower overall prevalence of Class III malocclusions, including 1.6% Italy, 1.6% Nigeria 1.4 Jordan [19,48].

In the Romanian orthodontic sample taken into study, a hypodivergent and normodivergent growth pattern was observed with equal frequency, while the mandibular angle most commonly presented a closed configuration, with a distribution similar to normal values. These results are consistent with reports indicating that Danish individuals tend to show more hypodivergent craniofacial characteristics compared to Korean populations, in line with previous research findings [30,49]. The length of the upper lip is also influenced by ethnicity; the Caucasian population, specifically the Romanian population, exhibits a shorter upper lip [50].

Our findings indicate a sex-related variation in terms of malocclusion patterns, skeletal anomalies, vertical growth patterns and upper incisors sagittal position. Thus, female patients exhibited the following features: a predominance of Class I and Class II division 2 malocclusion, a predominance of skeletal Class II associated with mandibular retrognathia, hyperdivergent vertical facial patterns and retroclined upper incisors. The tendency toward retroclination of the upper incisors observed in female patients may reflect their higher prevalence of Class II dental and skeletal patterns, possibly indicating a compensatory dentoalveolar mechanism. Our results are supported by Lone IM et al. (2023) and Alhammadi et al. (2022), who demonstrated that patients with skeletal Class II patterns often exhibit reduced maxillary incisor proclination or relative retroclination as a dentoalveolar adaptation to sagittal discrepancies, contributing to the camouflage of skeletal disharmony [15,51]. In addition, our results are also consistent with Steiner’s (1953) and Stahl et al. (2008) observations, who indicated that incisor inclination and position are dependent on the sagittal relationship between the maxilla and mandible [52,53]. This finding suggests that dental compensation in severe Class II malocclusion is predominantly influenced by the maxillary incisors.

Moreover, in a study published by Pillai et al. (2018), sex-related differences were also statistically significant in cephalometric analysis, with females exhibiting significantly lower cephalometric measurements [54].

Regarding the vertical facial pattern observed in Romanian orthodontic female subjects, the findings reported by Paddenberg-Schubert E et al. (2025) differ from ours, as female adult patients in their study exhibited a more horizontal growth pattern according cu Gonion angle, although the facial axis indicated a slightly more vertical tendency in Class II cases [55].

Furthermore, Romanian male orthodontic patients showed the following characteristics: a higher prevalence of Class III malocclusion and a skeletal Class III due to prognathic mandible, a normodivergent vertical facial pattern and proclined upper incisors. The tendency toward proclination of the upper incisors observed in males may reflect their higher prevalence of Class III dental and skeletal patterns, potentially indicating a compensatory dentoalveolar mechanism, as emphasized in recent studies [56]. Thus, our findings are in agreement with previously published studies reporting statistically significant differences between sexes [40,57].

The cephalometric analysis of orthodontic patients with Class II Division 1 malocclusion included in our study revealed a predominance of female subjects and highlighted a multifactorial skeletal and dentoalveolar pattern. The skeletal discrepancy was primarily characterized by mandibular retrognathism, as evidenced by the high prevalence of decreased SNB values and increased ANB angles, while maxillary positioning remained largely within normal limits in a considerable proportion of cases. Dentally, the condition was consistently associated with proclined maxillary incisors and, to a lesser extent, the mandibular incisors, contributing to the frequently observed reduction in the interincisal angle. From a vertical perspective, most patients exhibited normodivergent growth patterns, suggesting that sagittal discrepancies rather than vertical disproportions play a dominant role in this malocclusion type. Additionally, the tendency toward a closed mandibular angle and the high frequency of convex facial profiles further support the predominance of skeletal Class II characteristics driven by mandibular deficiency. These findings are consistent with previous reports and emphasize the importance of comprehensive cephalometric evaluation for accurate diagnosis and treatment planning, particularly in distinguishing between skeletal and dentoalveolar components of Class II Division 1 malocclusion.

The cephalometric characteristics identified in orthodontic patients with Class II Division 2 malocclusion in the present study indicate a clear predominance of female subjects and a distinct skeletal and dentoalveolar pattern. The skeletal discrepancy was mainly associated with mandibular retrognathism, as reflected by the high prevalence of decreased SNB values and universally increased ANB angles, while the maxilla was generally positioned within normal limits. A defining feature of this malocclusion type was the consistent retroclination of the maxillary incisors, accompanied by a marked increase in the interincisal angle, and, in over half of the cases, compensatory proclination of the mandibular incisors. From a vertical standpoint, a tendency toward hypodivergence was observed, supported by decreased FMA and S-N/Go-Gn angles and further reinforced by the universal presence of a closed mandibular angle, suggesting a short-face growth pattern. Additionally, the high frequency of convex facial profiles underscores the sagittal imbalance primarily driven by mandibular deficiency. These findings align with existing literature and highlight the importance of differentiating Class II Division 2 from Division 1 malocclusion, particularly given its characteristic dental compensation and reduced vertical dimensions, which have direct implications for individualized orthodontic treatment planning.

In the present study, the SNA angle was significantly different in Class II patients: patients with Class II/2 malocclusion exhibited higher SNA values than patients with Class II/1 malocclusion. This finding is inconsistent with the results reported by Jacob et al. (2014) [58].

The cephalometric evaluation of patients with Class III malocclusion in the present study revealed a predominance of male subjects and a heterogeneous skeletal pattern. The sagittal relationship was variably associated with both Class I and Class III skeletal configurations, most frequently driven by mandibular prognathism, as indicated by the increased SNB values observed in over half of the patients, alongside decreased or normal ANB angles. Maxillary positioning was more variable, with a considerable proportion of patients exhibiting normal SNA values, while others demonstrated a degree of maxillary retrusion. Dentally, a tendency toward proclination of the maxillary incisors was noted, likely representing a compensatory mechanism, whereas the mandibular incisors were most commonly normally inclined or retroclined. This dentoalveolar compensation is further reflected in the increased interincisal angle observed in half of the sample. From a vertical perspective, both hypodivergent and normodivergent growth patterns were identified, with a predominance of normal vertical relationships based on the S-N/Go-Gn angle. The mandibular angle was similarly variable, though most frequently within normal limits. In terms of facial profile, a balanced profile was most commonly observed, closely followed by a concave profile, consistent with the underlying skeletal Class III pattern. These findings highlight the complexity and variability of Class III malocclusion and emphasize the necessity of comprehensive cephalometric assessment to distinguish between skeletal and dentoalveolar components, which is essential for accurate diagnosis and the selection of appropriate orthodontic or orthopedic treatment strategies.

Regarding the growth pattern and the sagittal relationship between jaws, our findings are in agreement with those reported by Midlej et al. (2024), who described a predominantly horizontal growth pattern and mandibular prognathism, as reflected by an increased SNB angle value [59].

The limitations of this study refer to the fact that all included patients were Caucasians, mostly over 12 years old, which restricts the generalizability of the findings. Romanian orthodontic patients were included to minimize ethnic variation in cephalometric characteristics. In addition, estimating malocclusion prevalence based on sex-specific or orthodontic clinic samples may overestimate its occurrence, thereby reducing external validity. Future studies should use population-based samples to minimize selection bias.

## 5. Conclusions

The Romanian orthodontic patients are characterized by a predominance of Class I dental occlusion and Class I skeletal pattern, followed by Class II Division 1 and Class III malocclusions. Class II Division 1 was mainly associated with mandibular retrognathism and incisor proclination, whereas Class II Division 2 showed mandibular deficiency with maxillary incisor retroclination, increased interincisal angle, and a hypodivergent, short-face tendency. Class III malocclusion was more variable, most commonly involving mandibular prognathism with dentoalveolar compensation and concave or balanced profiles. Overall, a normal SNA angle was most frequent, SNB was mainly decreased, and ANB was increased, with proclined incisors, similar frequencies of hypo- and normodivergent patterns, and a predominantly closed mandibular angle.

Sex-related differences were evident, with Class II malocclusions affecting females more frequently, Class III malocclusions affecting males, and females showing lower cephalometric values and significant inter-sexual differences. The observed cephalometric differences between Class I, II and III malocclusions provide clinically relevant markers in vertical, sagittal, and dental dimensions that may provide descriptive reference data for similar orthodontic samples. Additionally, specific population and sex-related variations highlight the need to consider sex-specific characteristics in orthodontic diagnosis and treatment planning.

## Figures and Tables

**Table 1:** Clinical norm and interpretation of several cephalometric parameters.

Cephalometric Parameter	Definition	Clinical Norm	Interpretation	
			Decreased	Increased
SNA (°)	Angle formed by connecting the Sella-Nasion plane to the A point; determines the antero-posterior relationship of the maxilla to the anterior cranial base (S-N);	82° ± 2° (Mc Laughlin)	Retrognathic maxilla	Prognathic maxilla
SNB (°)	Angle formed by connecting the Sella-Nasion plane to the B point; determines the antero-posterior relationship of the mandible to the anterior cranial base (S-N);	80° ± 2° (Mc Laughlin)	Retrognathic mandible	Prognathic mandible
ANB (°)	Angle formed by subtracting SNB angle from SNA angle; determines a relative position of A point to B point, the sagittal jaw relationship;	2° ± 2° (Mc Laughlin)	Skeletal class III	Skeletal class II
S-N/Go-Gn (°)	Angle formed by the anterior cranial base (S-N) and the mandibular plane (Go-Gn); determines the inclination of the mandibular plane to the anterior cranial base;	32° ± 5° (Mc Laughlin)	Horizontal growing, counter-clockwise rotation, tendency to deep-bite	Vertical growing, clockwise rotation, tendency to open-bite
N-A-Pog (°)	Angle formed by Nasion-Point A plane and point A—Pogonion plane; it determines the angle of convexity (the convexity of the profile);	0° ± 5°	Concave profile	Convex profile
Interincisal (°)	Angle formed by the axes of the upper and lower incisors, important for the stability of the teeth; it describes the vertical and horizontal dimensions of the occlusion of the incisors;	135° ± 5° (Downs)	Proclined upper and lower incisors	Retroclined upper and lower incisors
Max1-FH (°)	Angle formed by the axis of the most prominent upper central incisor and the Frankfurt horizontal; it determines the inclination of the upper incisors relative to the Frankfurt horizontal plane;	110° ± 2° (Downs)	Retroclined upper incisors	Proclined upper incisors
Ar-Go-Me (°)	Angle formed by the reference lines Ar-Go and Go-Me; it determines the gonial angle, referring to the relation between the corpus of the mandible and the mandibular ramus;	130° ± 7° (Jarabak)	Anterior rotation of the mandible, vertical growth of the condyles	Posterior rotation of the mandible, posterior growth of the condyles
FMA (°)	Angle formed by the mandibular plane and the Frankfurt horizontal plane; it determines the divergency of the face;	24° (Tweed)	hypodivergent	hyperdivergent
IMPA (°)	Angle formed by the mandibular plane and the axis of the most prominent lower incisors; it determines the inclination of the lower incisors;	90° (Tweed)	Retroclined lower incisors	Proclined lower incisors

**Table 2:** Clinical and cephalometric characteristics of the orthodontic patients.

Parameter	Value
**Age** (Mean ± SD, Median (IQR))	20.07 ± 8.63, 16.65 (14.1–23.9)
**Age category** (No., %)	
8–12 years	30 (6.7%)
13–18 years	228 (50.7%)
≥18 years	192 (42.7%)
**Sex** (No., %)	
Female	261 (58%)
Male	189 (42%)
**Dental malocclusion** (No., %)	
Class I	228 (50.7%)
Class II	21 (4.7%)
Class II/1	120 (26.7%)
Class II/2	21 (4.7%)
Class III	60 (13.3%)
**SNA angle** (Mean ± SD, Median (IQR))	82.79 ± 3.98, 82.6 (79.7–85.5)
**ANB angle** (Mean ± SD, Median (IQR))	4.16 ± 6.82, 4.15 (1.5–6.1)
**SNB angle** (Mean ± SD, Median (IQR))	78.62 ± 7.79, 78.75 (75.8–82.1)
**S-N/Go-Gn angle** (Mean ± SD, Median (IQR))	32.31 ± 6.35, 32.6 (27.6–36.8)
**Skeletal anomaly** (No., %)	
Class I	195 (43.3%)
Class II—prognathic maxilla	78 (17.3%)
Class II—retrognathic mandible	129 (28.7%)
Class III—retrognathic maxilla	9 (2%)
Class III—prognathic mandible	39 (8.7%)
**N-A-Pog angle** (Mean ± SD, Median (IQR))	5.68 ± 7.87, 6.45 (0.7–11)
**Interincisal angle** (Mean ± SD, Median (IQR))	131.6 ± 12.77, 130.2 (123–138)
**Max1-FH angle** (Mean ± SD, Median (IQR))	114.46 ± 9.6, 114.8 (109–119)
**Ar-Go-Me angle** (Mean ± SD, Median (IQR))	125.07 ± 7.2, 125 (121–129)
**FMA angle** (Mean ± SD, Median (IQR))	22.96 ± 5.81, 22 (19–27)
**IMPA angle** (Mean ± SD, Median (IQR))	90.96 ± 8.65, 91 (86–98)

**Table 3:** Distribution of the patients according to dental malocclusion and age.

Age/Dental Malocclusion	8–12 Years		13–18 Years		≥18 Years		*p* *
	Nr.	%	Nr.	%	Nr.	%	
Class I	3	10%	129	56.6%	96	50%	**<0.001**
Class II	0	0%	9	3.9%	12	6.3%	
Class II/1	9	30%	57	25%	54	28.1%	
Class II/2	0	0%	21	9.2%	0	0%	
Class III	18	60%	12	5.3%	30	15.6%	

* Fisher’s Exact Test.

**Table 4:** Correlation between cephalometric measurements (dental and skeletal malocclusion, FMA angle, Max1-FH angle) and patients’ sex.

Dental Malocclusion	Female		Male		*p* *
	No.	%	No.	%	
I	144	55.2%	84	44.4%	<0.001
II	15	5.7%	6	3.2%	
II/1	69	26.4%	51	27%	
II/2	18	6.9%	3	1.6%	
III	15	5.7%	45	23.8%	
**Skeletal anomaly**					*p* *
I	105	40.2%	90	47.6%	<0.001
II-prognathic maxilla	45	17.2%	33	17.5%	
II-retrognathic mandible	96	36.8%	33	17.5%	
III-retrognathic maxilla	3	1.1%	6	3.2%	
III-prognathic mandible	12	4.6%	27	14.3%	
**FMA angle**					*p* *
Low	102	39.1%	84	44.4%	<0.001
Normal	99	37.9%	90	47.6%	
High	60	23%	15	7.9%	
**Max1-FH angle**					*p* *
Low	51	19.5%	18	9.5%	0.003
Normal	96	36.8%	63	33.3%	
High	114	43.7%	108	57.1%	

* Fisher’s Exact Test.

**Table 5:** Distribution of the patients according to dental malocclusion, age and gender.

Age/Dental Malocclusion	<18 years		≥18 years		*p* *
	Nr.	%	Nr.	%	
Class I	132	51.2%	96	50%	0.457
Class II	96	37.2%	66	34.4%	
Class III	30	11.6%	30	15.6%	
**Gender/Malocclusion**	**Female**		**Male**		***p*** *
	**Nr.**	**%**	**Nr.**	**%**	
Class I	144	55.2%	84	44.4%	<0.001
Class II	102	39.1%	60	31.7%	
Class III	15	5.7%	45	23.8%	

* Fisher’s Exact Test.

**Table 6:** Multivariable multinomial logistic regression model used in the prediction of dental malocclusion using age and gender.

Group *	Parameter	OR (95% C.I.)	*p*
**Class II**	Female	0.996 (0.655–1.513)	0.984
	<18 years	1.058 (0.702–1.594)	0.789
**Class III**	Female	0.188 (0.099–0.360)	<0.001
	<18 years	0.648 (0.358–1.172)	0.152

* Class I—Reference group. Model parameters: χ^2^(4) = 33.673, *p* < 0.001, Goodness-of-Fit: Pearson—*p* = 0.824, Deviance—*p* = 0.823, Nagelkerke R^2^ = 0.084.

**Table 7:** Comparison of cephalometric measurements according to dental malocclusion.

Parameter	Class II/1	Class II/2	Class III	*p*
***Age (Median (IQR))***	16.8 (14.5–28.5)	15 (14–17.2)	17.5 (10.6–21.9)	0.015 *
***Age group (Nr., %)***	**Class II/1**	**Class II/2**	**Class III**	***p***
8–12 years	9 (7.5%)	0 (0%)	18 (30%)	<0.001 **
13–18 years	57 (47.5%)	21 (100%)	12 (20%)	
≥18 years	54 (45%)	0 (0%)	30 (50%)	
***Gender (Nr., %)***				
Female	69 (57.5%)	18 (85.7%)	15 (25%)	<0.001 **
Male	51 (42.5%)	3 (14.3%)	45 (75%)	
***Skeletal anomaly (Nr., %)***				
Class I	30 (25%)	0 (0%)	27 (45%)	<0.001 **
Class II—prognathic maxilla	30 (25%)	6 (28.6%)	0 (0%)	
Class II—retrognathic mandible	60 (50%)	15 (71.4%)	0 (0%)	
Class III—retrognathic maxilla	0 (0%)	0 (0%)	6 (10%)	
Class III—prognathic mandible	0 (0%)	0 (0%)	27 (45%)	
**Analyzed angles (Median (IQR))**				
***SNA***	81.1 (79.4–84.5)	82.9 (81.2–86.8)	81.6 (79.5–85.1)	0.033 *
***ANB***	5.95 (4.1–7.57)	6.7 (4.6–7.2)	−0.35 (−3–0.78)	<0.001 *
***SNB***	76 (73.8–78.6)	76.8 (75.8–79.2)	83.05 (79.4–88.6)	<0.001 *
***S-N/Go-Gn***	32.4 (29–36.6)	26.1 (21.4–34.1)	31.8 (27–36.6)	0.001 *
***N-A-Pog***	9.4 (4.4–14.4)	10.9 (7.6–13.9)	−2.4 (−11.3–0.7)	<0.001 *
***Interincisal***	124 (116–131)	148 (137–158)	135 (124–145)	<0.001 *
***Max1-FH***	117 (113–120)	102 (90–103)	118 (110–125)	<0.001 *
***Ar-Go-Me***	123 (121–126)	114 (110–121)	127 (124–134)	<0.001 *
***FMA***	23 (21–26.75)	19 (12–24)	22.5 (18.2–26.7)	0.002 *
***IMPA***	94 (89–99.75)	95 (91–98)	86 (77.5–91.5)	<0.001 *

* Kruskal–Wallis H Test, ** Fisher’s Exact Test.

## Data Availability

The data presented in this study are available on request from the corresponding authors. The data are not publicly available due to privacy reasons.
